# A molecular study of *Neophyllaphisvaricolor* (Hemiptera, Aphididae) in Costa Rica

**DOI:** 10.3897/zookeys.865.35213

**Published:** 2019-07-22

**Authors:** Adonay Zúñiga-Centeno, Izayana Sandoval-Carvajal, Mauricio Montero-Astúa, William Villalobos-Muller, Nguyễn Bảo Quốc, Nicolás Pérez Hidalgo

**Affiliations:** 1 Universidad de Costa Rica, Escuela de Agronomía, San José, 11501-2060, Costa Rica; 2 Universidad de Costa Rica, Centro de Investigación en Biología Celular y Molecular, San José, 11501-2060, Costa Rica; 3 Nong Lam University, Research Institute for Biotechnology and Environment, Ho Chi Minh City, 700000, Vietnam; 4 Instituto de Biología Integrativa de Sistemas (I2SysBio), Centro Mixto Universidad de Valencia-CSIC, Valencia, 46980, España

**Keywords:** Aphids, cytochrome *c* oxidase subunit I, DNA barcoding, elongation factor I, integrative taxonomy, phylogenetic analysis, *
Podocarpus
*

## Abstract

The genus *Neophyllaphis* (Takahashi) (Aphididae: Neophyllaphidinae) is composed of 18 species; however, in the Americas only nine species have been reported previously. A new species, *Neophyllaphisvaricolor* Miller & Halbert, was described in 2014 in USA. Colonies resembling those of this new species have been observed in Costa Rica on *Podocarpus* spp. In order to determine if *N.varicolor* is also present in Costa Rica, we sampled *Neophyllaphis* colonies from *Podocarpusfalcatus* and *P.chinensis*. Additionally, we sampled individuals from *Podocarpus* sp. in Spain and Vietnam. DNA of each sample was extracted and used to amplify and sequence the cytochrome *c* oxidase subunit I (COI) and elongation factor I (EF-1α) partial regions. According to morphological characteristics, sequences comparisons done in GenBank and BOLD, and phylogenetic analyses, the colonies collected from *Podocarpus* spp. in Costa Rica and the colony from Vietnam corresponded to the species *N.varicolor*. To the best of our knowledge this is the first report of the presence of *N.varicolor* in Central America and Vietnam.

## Introduction

*Neophyllaphis* Takahashi (Aphididae: Neophyllaphidinae) is a genus occurring predominantly in the southern hemisphere and composed of 18 species characterized by a body covered with pulverulence and waxy secretions, antenna with a short terminal process, siphuncular pores on small cones, cauda with a constriction in the middle and with a caudal knob, and annular secondary rhinaria that are only present in alatae (Quednau 2010). The species of this genus live on species of Podocarpaceae, Araucariaceae, Myrtaceae, and less frequently on Cupressaceae (Hales and Lardner 1988; Miller and Halbert 2014). The genus is divided into two subgenera, a nominotypical subgenus Neophyllaphis, and the subgenus Chileaphis Essig, 1953 (Hille Ris Lambers 1967). Species of the subgenus Chileaphis have a very restricted distribution in South America while the subgenus Neophyllaphis is distributed in temperate and tropical regions of Africa, Asia, and Australia, and some species have been introduced in North America (Hille Ris Lambers 1967; Russell 1982; Qiao et al. 2001; Blackman and Eastop 2019). In Europe, only the species *Neophyllaphispodocarpi* Takahashi 1920 has been reported (Aguiar et al. 2013; Pérez Hidalgo et al. 2015).

In the Americas there are nine *Neophyllaphis* species (Mier Durante et al. 2008; Quednau 2010; Miller and Halbert 2014; Blackman and Eastop 2019), six species in the subgenus Chileaphis and three invasive species in the subgenus *Neophyllaphis: N.araucariae* Takahashi 1937, *N.podocarpi* (Mier Durante et al. 2008; Quednau 2010), and the newest described as *N.varicolor*Miller and Halbert 2014. The species *N.podocarpi* and *N.varicolor* have been reported in the western hemisphere only in the United States. Both species have been recorded in Florida and Louisiana, while only *N.podocarpi* has been found in California, Mississippi, and Texas (Russell 1982; Skvarla et al. 2017). The species *N.araucariae* is the only one living on the genus *Araucaria* Juss. and it is native of the Oriental region (perhaps native to Norfolk Island or Australia). There are reports of *Neophyllaphisaraucariae* in the United States (Florida, California, Hawaii) (Timberlake 1917; Russell 1982), Mexico (Peña-Martínez 1985), Panama (Russell 1982), Venezuela (Cermeli 1990) and Costa Rica (Voegtlin et al. 2003). In Costa Rica, it is the only reported species of *Neophyllaphis* (Voegtlin et al. 2003; Villalobos Muller et al. 2010).

**Figure 1.**
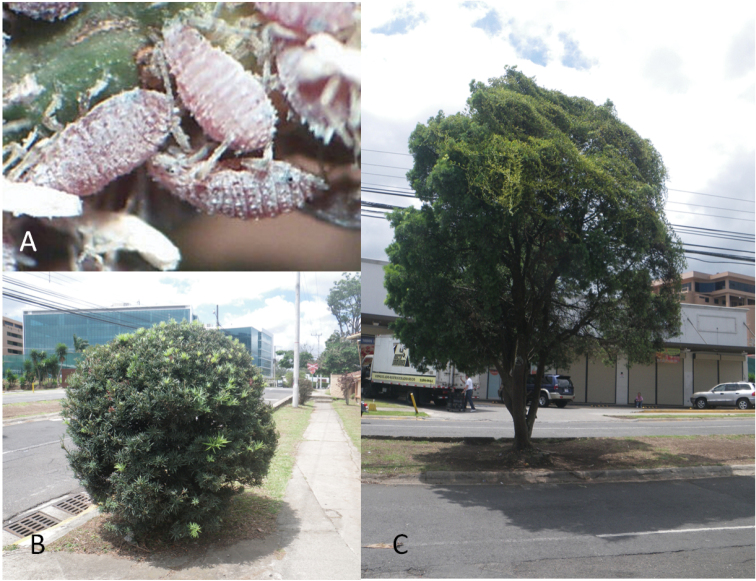
**A***Neophyllaphisvaricolor* Miller & Halbert, apterous individuals from Costa Rica **B***Podocarpuschinensis* Wall. ex J.Forbes, and **C***Podocarpusfalcatus* (Thunb.) Mirb.

The newly recorded species *N.varicolor*, described by Miller and Halbert (2014), is characterized by a dorsoventrally flattened body and color variations of individuals in the same colony that may be yellow, orange, red or purple. The species was noticed beginning in 2010 in different counties in Florida. There are no records from outside USA. Multi-colored aphid colonies resembling those described by Miller and Halbert (2014), were found infesting the trees *Podocarpusfalcatus* (Thunb.) Mirb. and *P.chinensis* Wall. ex J.Forbes Wall. in Costa Rica during 2014 (Figure 1). Morphological identification and molecular analyses were done to determine if the new species, *N.varicolor*, is also present in Costa Rica.

## Materials and methods

### Sample collection

Thirteen aphid colonies were sampled for this study. Nine samples from colonies on *Podocarpus* spp. L’Hér.ex Pers. were collected from different localities in San José province and samples from two colonies of *N.araucariae* were collected on *Araucaria* spp. in San José and Cartago provinces (Costa Rica). Additionally, two samples, one of *N.podocarpi* from Gerona (Spain) and one *Neophyllaphis* sp. multicolored colony from a *Podocarpus* sp. shrub in Vietnam were collected for comparison (Table 1). Samples were maintained at -35 °C in 95% ethanol for molecular analyses and at 6 °C in 70% ethanol for morphological analysis.

**Table 1.** Samples of *Neophyllaphis* spp. colonies (individual aphids per colony indicated by a, b, and c) and the corresponding accession numbers of COI and EF-1α sequences deposited at GenBank.

**Species**	**Colony code**	**Collection place**	**Host plant**		**Accession numbers**	
					**COI**	**EF-1α**
* N. varicolor *	CR14-002	Montes de Oca, San José (9.935764°N, 84.057778°W)	*Podocarpusfalcatus* (Thunb.) Mirb.	a	MK174294	ND
				b	MK174295	MK174326
				c	MK174296	MK174327
* N. varicolor *	CR14-004	Montes de Oca, San José (9.934636°N, 84.058056°W)	*Podocarpuschinensis* Wall. ex J.Forbes	a	MK174297	ND
				b	MK174298	ND
				c	MK174299	MK174328
* N. varicolor *	CR14-005	Montes de Oca, San José (9.934567°N, 84.059167°W)	*Podocarpusfalcatus* (Thunb.) Mirb.	a	MK174300	ND
				b	MK174301	MK174329
				c	MK174302	ND
* N. varicolor *	CR14-012	Goicoechea, San José (9.946283°N, 84.053056°W)	*Podocarpusfalcatus* (Thunb.) Mirb.	a	MK174303	MK174330
				b	MK174304	ND
				c	MK174305	ND
* N. varicolor *	CR14-013	Curridabat, San José (9.923417°N, 84.033056°W)	*Podocarpusfalcatus* (Thunb.) Mirb.	a	MK174306	MK174331
				b	MK174307	ND
				c	MK174308	ND
* N. varicolor *	CR14-033	Montes de Oca, San José (9.943450°N, 84.018889°W)	*Podocarpuschinensis* Wall. ex J.Forbes	b	MK174309	ND
				c	MK174310	MK174332
* N. varicolor *	CR14-034	Montes de Oca, San José (9.939783°N, 84.009444°W)	*Podocarpusfalcatus* (Thunb.) Mirb.	a	MK174311	ND
				b	MK174312	MK174333
				c	MK174313	ND
* N. varicolor *	CR14-127	San José, San José (9.929947°N, 84.070278°W)	*Podocarpusfalcatus* (Thunb.) Mirb.	a	MK174314	ND
				b	MK174315	ND
				c	MK174316	MK174334
* N. varicolor *	CR14-295	Vázquez de Coronado, San José (9.969086°N, 84.016944°W)	*Podocarpusfalcatus* (Thunb.) Mirb.	a	MK174317	MK174335
				b	MK174318	ND
				c	MK174319	ND
* N. araucariae *	CR14-364	Paraíso, Cartago (9.852750°N, 83.807500°W)	*Araucariaaraucana* (Molina) K. Koch	b	MK174320	MK174336
				c	MK174321	MK174337
* N. podocarpi *	CR14-398	Gerona, Spain (41.676944°N, 2.801944°W)	*Podocarpusneriifolius* D.Don	c	MK174325	MK174341
* N. varicolor *	CR14-397	Long Dinh, Vietnam (10.384510°N, 106.166800°W)	*Podocarpus* sp. L’Hér.ex Pers.	a	MK463550	MK463553
				b	MK463551	MK463554
				c	MK463552	MK463555
* N. araucariae *	CR14-423	Vázquez de Coronado, San José (9.970153°N, 84.030833°W)	*Araucariaheterophylla* (Salisb.) Franco	a	MK174322	MK174338
				b	MK174323	MK174339
				c	MK174324	MK174340

### Morphological identification

Individuals preserved in 70% ethanol were cleared using KOH and acetic acid and mounted in slides with Canada balsam. The morphological identification of the specimens was done using a Leica Z16 microscope. We measured structures with taxonomic value and used the keys from Miller and Halbert (2014) and Blackman and Eastop (2019) to identify species of *Neophyllaphis*. The photographs were taken with a Leica Z16 microscope, equipped with a CF500 camera and LAS 4.9 (Leica) image capture. Mounted specimens were deposited at the aphid collection of the Instituto de Biología Integrativa de Sistemas (Centro Mixto Universidad de Valencia-CSIC, Spain) and in the Centro de Investigación en Biología Celular y Molecular (CIBCM), Universidad de Costa Rica.

### DNA extraction and molecular identification

DNA was extracted from three individual aphid specimens per colony (preserved in 95% ethanol) using the animal tissue protocol of NucleoSpin Tissue extraction kit (Macherey-Nagel, Germany) following the manufacturer instructions with a modification at the elution step; it was made by duplicate, using 50 µL of elution buffer each time.

For the molecular identification and phylogenetic analysis of the *Neophyllaphis* spp. samples, we amplified the cytochrome *c* oxidase subunit I (COI) and the elongation factor 1α (EF-1α) genes. To amplify COI we used the primer pair C1-J-1490 (= LepF) and C1-N-2198 (= LepR) to obtain an amplicon of 658 bp (Hajibabaei et al. 2006, Miller and Halbert 2014), and the primers C1-J-1718 (Simon et al. 1994) and C1-J-2411 (Lagos et al. 2012) to obtain an amplicon of 868 bp. The EF-1α gene was amplified using the primers EF-3 and EF-6 to generate a fragment of 785 bp (Miller and Halbert 2014). All PCR reactions were done in a final volume of 25 μL with final concentration of 1X Dream Taq Master Mix (2X, Thermo Scientific, Lithuania), 200 nM of each primer, 1% trehalose dehydrated, and 5 µL of DNA. Reactions were run with the following thermocycle profiles: 94 °C x 1 min; 5 x (94 °C x 40 s, 45 °C x 40 s, 72 °C x 1 min); 35 x (94 °C x 40 s, 51 °C x 40 s, 72 °C x 1 min); 72 °C x 5 min (Hajibabaei et al. 2005) for primer pair C1-J-1490 / C1-N-2198; and 96 °C x 2 min; 40 x (95 °C x 30 s, 53 °C x 30 s, 72 °C x 2 min); 72 °C x 10 min for primers C1-J-1718 / C1-J-2411 (Lagos et al. 2012).

Amplicons of COI and EF-1α were directly sequenced after purification in reverse and forward directions by the Sanger method (Macrogen, Korea). The final contigs were obtained using BIOEDIT 7.0 (Hall 1999) and were assigned preliminarily to a species by alignment using the BLAST tool of NCBI (Altschul et al. 1990) and the Identification Engine tool at BOLD (Ratnasingham and Hebert 2007). Sequences obtained are available in GenBank (Table 1).

### Phylogenetic analyses

Phylogenetic analyses of *Neophyllaphis* spp. samples were done using partial sequences of the COI gene. Additionally, partial sequences of COI (nucleotide positions from 94 to 570 according to the reference sequence KF199852) and EF-1α (nucleotide positions from 81 to 546 according to the reference sequence KF199851) were concatenated using BIOEDIT tool (Hall 1999) and a phylogenetic tree was generated. Phylogenetic analyses were done using a mixed model of Bayesian phylogenetic inference in MrBayes tool using a Markov Chain Monte Carlo (MCMC) search with ten million generations (Huelsenbeck and Ronquist 2001). The trees were visualized and edited using the tool FigTREE v1.4.2 (Rambaut and Drummond 2012).

Sequences obtained from GenBank (www.ncbi.nlm.nih.gov/Genbank) of the species *N.varicolor* (COI: KF199852; EF-1α: KF199851, USA), *N.podocarpi* (COI: EU701821, Japan and JQ920926, China), *Neophyllaphisbrimblecombei* Carver (COI: JF883870, Australia) and from BOLD Systems (www.barcodinglife.org) for *Neophyllaphistotarae* Cottier (COI: RFBAD211_08, New Zealand) were included for comparison in the phylogenetic analyses. Sequences of *Greenideaanonae* (Pergande) (COI: JQ926000; EF-1α: KF856808, China) and *Greenideapsidii* van der Goot (COI: JQ925937 and EF-1α: KF856814, China, and COI: EU701673, USA) were used as an outgroup because the genus *Greenidea* clustered relatively close to *Neophyllaphis* in a COI phylogeny (Foottit et al. 2008).

## Results

### Morphological identification

**Figure 2.**
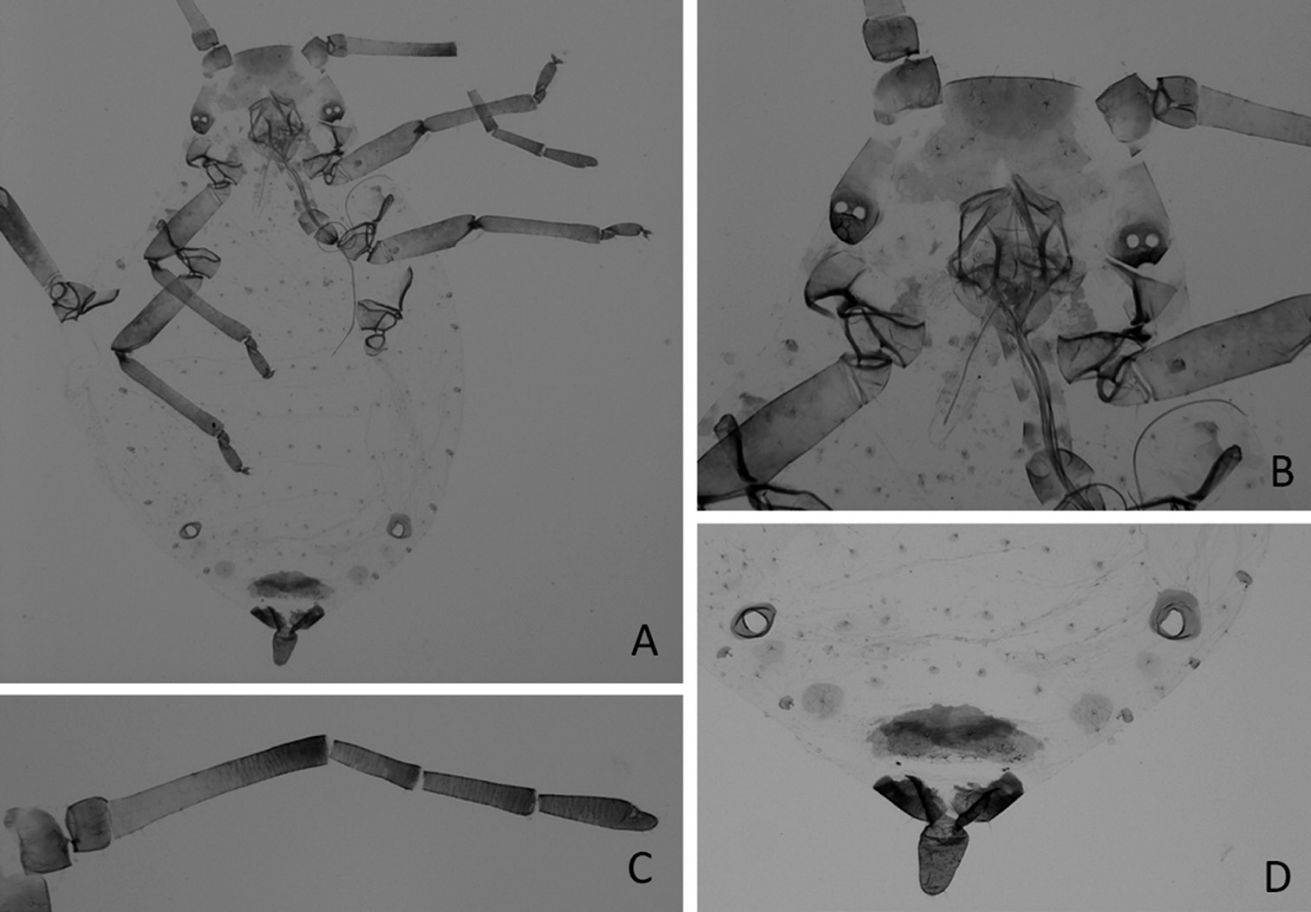
*Neophyllaphisvaricolor* Miller & Halbert, apterous **A** body **B** anterior part of the body **C** antennae, and **D** posterior part of body.

**Figure 3.**
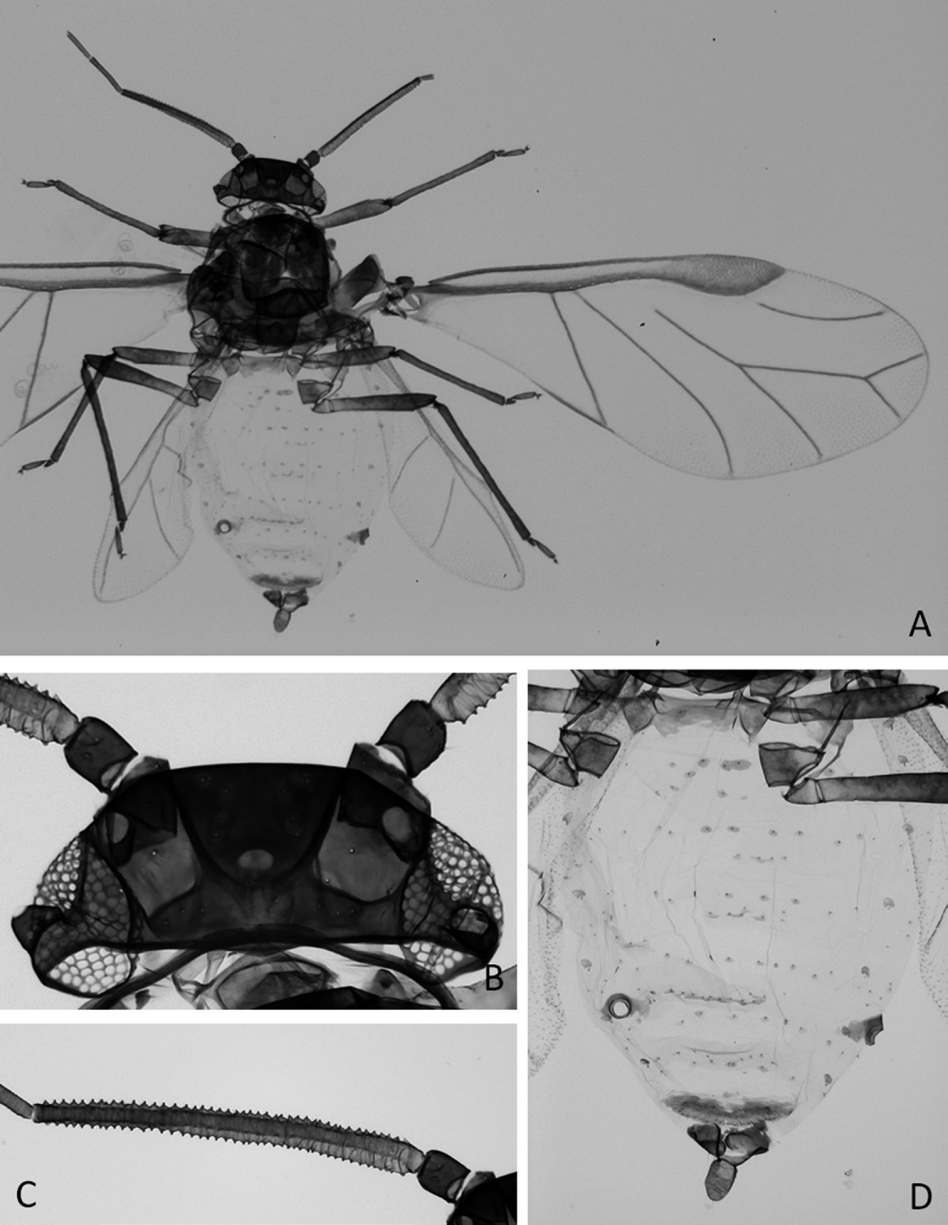
*Neophyllaphisvaricolor* Miller & Halbert, alate **A** alate aphid **B** head **C** antennal segment III and **D** abdomen.

The metric and meristic characters (including color when alive) of the approximately 70 apterous specimens (Figure 2) and of the 12 winged (Figure 3) of *Neophyllaphis* studied in Costa Rica and their comparison with the detailed description of *N.varicolor* by Miller and Halbert (2014), confirmed that the Costa Rican samples belong to this species described from North America. However, the Ant. III/Ant. IV ratios of our apterous specimens varied from 2.32 to 2.88 (·= 2.56). Thus, the antennal ratio character used to separate *Neophyllaphisfransseni* Hille Ris Lambers and *N.varicolor* (ratio greater than 2.6 in apterae) from *Neophyllaphisgingerensis* Carver, *N.totarae*, *N.brimblecombei*, *Neophyllaphislanata* Hales & Lardner and *N.podocarpi* (with a ratio shorter than 2.6), should be reevaluated (Miller and Halbert 2014; Blackman and Eastop 2019).

### Molecular identification and phylogenetic analysis

A total of 39 individual aphids was analyzed by molecular means; COI sequence data were obtained for 35 individuals and EF-1α sequences for 19 individuals. We did not obtain final sequences for all three aphid individuals processed per colony because amplifications failed for some individuals or the sequencing reaction rendered low quality reads. All COI and EF-1α sequences obtained from samples morphologically identified as *N.varicolor* corresponded to this species according to the identification made in BOLD systems with 99.5% (KF199852.1) and 99.7% (KF199851.1) of similarity, respectively. It was not possible to corroborate the identification of the species *N.araucariae* by sequence identity comparison because data for this species is not available at GenBank or BOLD.

All COI sequences of *N.varicolor* from Costa Rica were identical, while, EF-1α sequences showed 0.6% difference. Sequences of COI and EF-1α of *N.araucariae* did not show intraspecific variation. Interspecific genetic variation between *N.varicolor* and *N.araucariae* was of 11.4% in COI sequences and 13.4% in EF-1α sequences.

All 26 partial sequences of COI from individuals morphologically identified as *N.varicolor* were grouped within the same clade, together with the *Neophyllaphis* sp. sample from Vietnam and the reference sequence of *N.varicolor* (GenBank Acc. No. KF199852) from Florida. This clade clustered independently from available sequences for *N.araucariae*, *N.brimblecombei*, *N.podocarpi*, and *N.totarae*. Similarly, all COI sequences of *N.araucariae* were grouped in the same clade, supporting identifications by morphological characters for both species. The *N.araucariae* cluster showed more relatedness to *N.totarae* than to the clade comprising *N.podocarpi*, *N.brimblecombei*, and *N.varicolor*.

**Figure 4.**
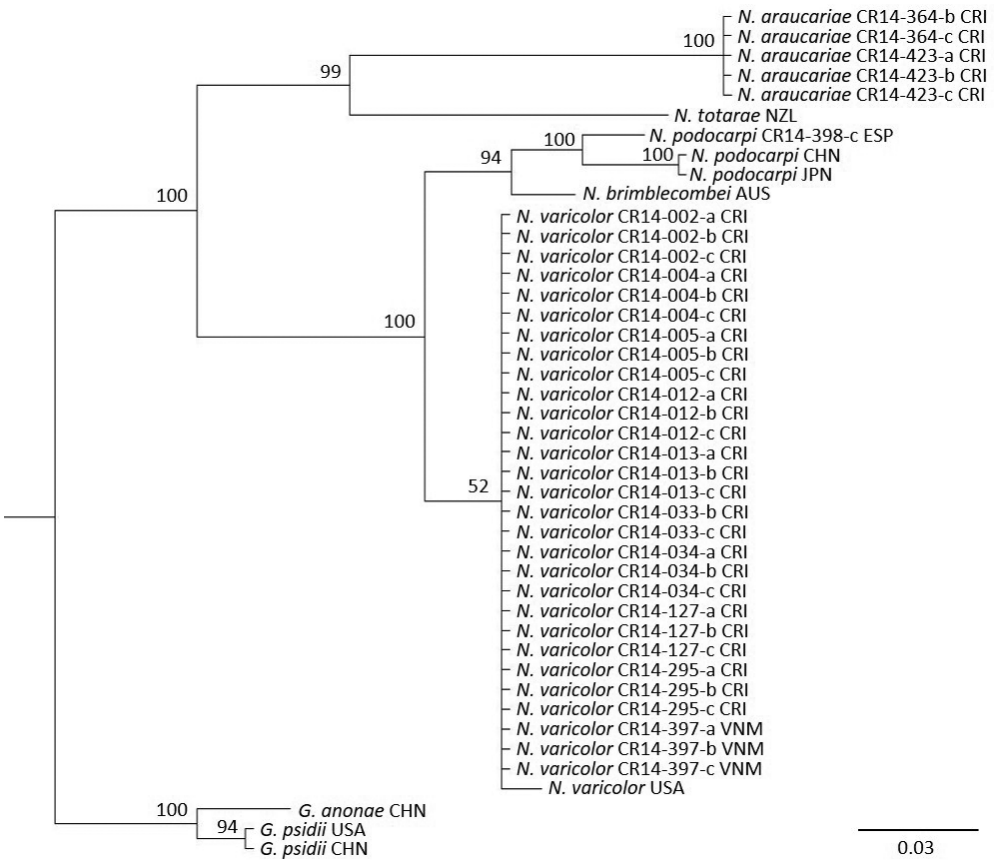
Phylogenetic analysis of *Neophyllaphisaraucariae* Takahashi, *Neophyllaphisbrimblecombei* Carver, *Neophyllaphispodocarpi* Takahashi, *Neophyllaphistotarae* Cottier, and *Neophyllaphisvaricolor* Miller & Halbert, using sequences of cytochrome c oxidase subunit I, made with Bayesian inference and using as outgroup *Greenideapsidii* van der Goot. and *Greenideaanonae* (Pergande). Sequence codes: species name - colony code - (a, b or c represent the specimen sampled) - country code. Key: AUS: Australia, CHN: China, CRI: Costa Rica, ESP: Spain, JPN: Japan, NZL: New Zealand, USA: United States, VNM: Vietnam. Scale bar represents 0.03 changes per site.

**Figure 5.**
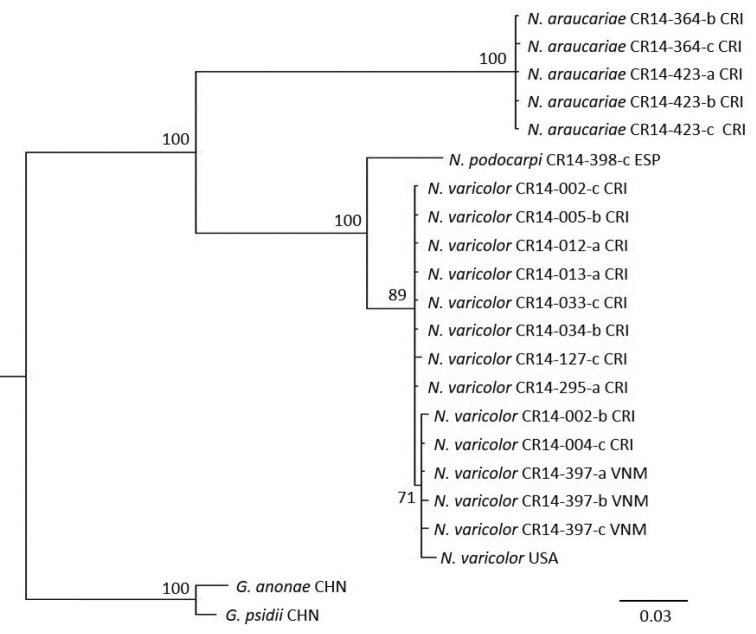
Phylogenetic analysis of *Neophyllaphisaraucariae* Takahashi, *Neophyllaphispodocarpi* Takahashi and *Neophyllaphisvaricolor* Miller & Halbert, using concatenated sequences of cytochrome *c* oxidase subunit I and elongation factor 1α made by Bayesian inference and using as outgroup *Greenideapsidii* van der Goot. and *Greenideaanonae* (Pergande). Sample names: species name - colony code - (a, b or c represent the specimen sampled) - country code. Key: CHN: China, CRI: Costa Rica, ESP: Spain, USA: United States, VNM: Vietnam. Scale bar represents 0.03 changes per site.

The phylogenetic analysis made with a concatenated sequence composed of partial COI and EF-1α sequences showed a clade grouping all the sequences of *N.varicolor* and another clade with the sequences of *N.araucariae*, in accordance with the COI phylogenetic tree (Figure 4) and with the morphological identification (Figure 5).

## Discussion

According to our results, the recently described aphid species *N.varicolor* also is present in Costa Rica and Vietnam. In addition to *N.varicolor* sequence information for Costa Rica and Vietnam, we also generated and submitted partial COI and EF-1α sequences for the species *N.araucariae* to GenBank for the first time. Indeed, we found few sequences available for the genus *Neophyllaphis* in public databases. Publicly available sequence information for all the describe species is important for comprehensive studies of the genus.

The morphological studies and molecular comparisons of COI and EF-1α sequences, supported the description of the new, distinct species, *N.varicolor* on *Podocarpus* spp. (Miller and Halbert 2014). All COI and EF-1α sequences of *N.varicolor* showed less than 0.5% of difference to the reference *N.varicolor* sequences deposited in GenBank. Previous studies have determined that the intraspecific variation in aphids is circa 0.6% in most of the species. There are some exceptions, like *Neomyzuscircunflexus* (Buckton), with 3.14%, the species with the highest intraspecific variation in COI out of 300 aphid species included in the study by Foottit et al. (2008).

The COI gene was characterized by a high interspecific variation (Floyd et al. 2009). The level of interspecific variation of COI (11.4%) and EF-1α (13.4 %) observed between sequences of *N.varicolor* and *N.araucariae* from Costa Rica, was congruent with the high genetic divergence expected between different species. However, in some cases, the interspecific variation of COI between congeneric species could be less than 1% (Chen et al. 2012).

According to our phylogenetic analyses made with COI region, the species *N.podocarpi* and *N.brimblecombei* are most related to *N.varicolor*, similar to findings by Miller and Halbert (2014); however, few sequences of *Neophyllaphis* species are available in GenBank or BOLD, so many species are not represented in the phylogenetic analysis. Previous studies have reported that *N.podocarpi* and *N.brimblecombei* have the same number of chromosomes and high morphological similarity, which suggest a recent separation between the species (Hales and Lardner 1988). Therefore, it is plausible to hypothesize that both species also were separated recently from *N.varicolor* in evolutionary time.

Our discoveries of *N.varicolor* in Costa Rica and Vietnam represent the first time that *N.varicolor* is reported outside of the USA. Currently, the genus *Neophyllaphis* is thus represented by two species in Costa Rica: *N.varicolor* and *N.araucariae*.

The presence in Vietnam of *N.varicolor* suggests that it is a species native to Southeast Asia. However, the genus *Neophyllaphis* presents taxonomic problems (Blackman and Eastop 2019) that must be solved with a good taxonomic, bionomic and molecular revisions. A full revision of the genus might shed better light on the geographic origins of the different species (Nibouche et al. 2018).
